# Poster Session I - A33 *HYMENOLEPIS DIMINUTA*-DERIVED EXTRACELLULAR VESICLES UPREGULATE A REGULATORY PHENOTYPE IN MURINE MACROPHAGES AND PROMOTE INTERACTION WITH T CELLS

**DOI:** 10.1093/jcag/gwaf042.033

**Published:** 2026-02-13

**Authors:** P Volk, L Kraemer, A Wang, D McKay

**Affiliations:** University of Calgary, Calgary, AB, Canada; University of Calgary, Calgary, AB, Canada; University of Calgary, Calgary, AB, Canada; University of Calgary, Calgary, AB, Canada

## Abstract

**Background:**

Extracellular vesicles (EVs) may participate in communication and immunoregulation in host-helminth interactions in the GI tract. Infection with the tapeworm *Hymenolepis diminuta* (Hd) has been shown to reduce the severity of chemical-induced colitis in mice: we hypothesized that Hd-EVs could play a role in this event. Thus, the ability of Hd-EVs to modulate macrophage activity and interact with T cells was assessed.

**Aims:**

To determine if Hd-EVs elicit a cytokine/chemokine response from murine macrophages and if Hd-EV treated macrophages influence T cell function.

**Methods:**

Adult *H. diminuta* were incubated in serum-free RPMI media for 6h and EVs in the isolated from media using the Exoeasy Isolation kit and dialyzed in a 10,000 MWCO cassette. Bone marrow-derived macrophages were isolated from C57Bl/6 mice and 2.5x10^5^ cells/mL were treated with 5 μg/mL protein Hd-EVs for 24h. Chemokine and cytokine expression was assessed by qPCR and ELISA, while PD-L1 and IL-4rα-chain expressions were measured by flow cytometry. Macrophages were cocultured with anti-CD3/CD28 activated CD4^+^ T cells at a 1:4 ratio for 120h and proliferation and Foxp3 expression assessed.

**Results:**

Hd-EV treated-macrophages (M(Hd-EVs)) displayed increased mRNA expression of CCL-2, -5 and -7, and increased CCL-5 protein (2.9±0.3 ng/mL; n = 4). Hd-EVs evoked a small increase in TNFα and a marked increase in IL-10 production by the macrophages (2.1±0.4 ng/ml; n = 6). These events were not observed with control, empty liposomes or 15 pg/ml LPS, the level of contamination in the Hd-EVs. Surface protein expressions of PD-L1 (93±1.4% positive, n = 3) and IL-4ra (93±1.7% positive, n = 3) were significantly increased in M(Hd-EVs). Splenic T cells in contact with M(Hd-EVs) proliferated and increased differentiation to Foxp3^+^ Tregs (49.4%±6.8 vs 25.1%±1.6 M(0), n = 3).

**Conclusions:**

Hd-EVs invoke a unique phenotype in murine macrophages, with increases in PD-L1 and IL-10 suggestive of a regulatory phenotype that could be reinforced or enhanced by IL-4 due to increased surface expression of IL-4rα. This is further supported by the increased number of Foxp3+ Tregs observed after M(Hd-EV)-CD4+ T cell coculture. While the functional significance of the M(Hd-EV) remains to be comprehensively defined, the data herein support the speculation that development of an M(Hd-EV) after infection with *H. diminuta* could directly, or indirectly via induction of regulatory T cells, contribute to the suppression of chemical-induced colitis.

**Funding Agencies:**

NRC

